# Bacterial and fungal components of the microbiome have distinct roles in Hawaiian *Drosophila* reproduction

**DOI:** 10.1093/ismeco/ycae134

**Published:** 2024-11-04

**Authors:** Matthew J Medeiros, Laura Seo, Aziel Macias, Donald K Price, Joanne Y Yew

**Affiliations:** Pacific Biosciences Research Center, University of Hawai`i at Mānoa, 1993 East West Rd., Honolulu, HI 96826, United States; School of Life Sciences, University of Nevada, Las Vegas, 4505 S Maryland Pkwy, Las Vegas, NV 89154-4004, United States; School of Life Sciences, University of Nevada, Las Vegas, 4505 S Maryland Pkwy, Las Vegas, NV 89154-4004, United States; School of Life Sciences, University of Nevada, Las Vegas, 4505 S Maryland Pkwy, Las Vegas, NV 89154-4004, United States; School of Life Sciences, University of Nevada, Las Vegas, 4505 S Maryland Pkwy, Las Vegas, NV 89154-4004, United States; Pacific Biosciences Research Center, University of Hawai`i at Mānoa, 1993 East West Rd., Honolulu, HI 96826, United States; School of Life Sciences, University of Nevada, Las Vegas, 4505 S Maryland Pkwy, Las Vegas, NV 89154-4004, United States

**Keywords:** bacterial-fungal interactions, Hawaiian *Drosophila*, oogenesis, lipids, reproduction

## Abstract

The microbiome provides numerous physiological benefits for host animals. The role of bacterial members of microbiomes to host physiology is well-documented. However, much less is known about the contributions and interactions of fungal members, even though fungi are integral components of many microbiomes, including those of humans and insects. Here, we used antibacterial and antifungal drugs to manipulate the gut microbiome of a Hawaiian picture-wing *Drosophila* species, *Drosophila grimshawi*, and identified distinct effects for each treatment on microbiome community stability, reproduction, and lipid metabolism. Female oogenesis, fecundity, and mating drive were significantly diminished with antifungal treatment. In contrast, male fecundity was affected by antibacterial but not antifungal treatment. For males and females, simultaneous treatment with both antibacterial and antifungal drugs resulted in severely reduced fecundity and changes in fatty acid levels and composition. Microbial transplants using frass harvested from control flies partially restored microbiome composition and female fecundity. Overall, our results reveal that antibacterial and antifungal treatments have distinct effects on host fecundity, mating behavior, and lipid metabolism, and that interkingdom interactions contribute to microbial community stability and reproduction.

## Introduction

The gut microbiome functions as a “virtual” organ for many animals, facilitating diverse physiological functions including the provision of essential nutrients, immunoprotection, detoxification, and energy metabolism [1, 2]. In particular, the contributions of bacteria have been studied intensely in animal hosts from humans to insects [3]. However, fungi are also significant constituents of many animal microbiomes [4, 5]. The diversity of pathogenic fungal lifestyles and infection strategies has been extensively documented [6, 7]. In the case of the host-specific pathogens *Ophiocordyceps* and *Entomopththora muscae,* fungi create “zombies” of insect hosts in order to induce climbing behavior and increase fungal spore [8–10].

Fewer beneficial roles for the mycobiome, the fungal component of the microbiome, have been identified. Fungi enhance the immune system in mice [11], offer protection from pathogenic bacteria, and assist in nutritional scavenging [4]. For many species of drosophilids, yeast serve as a source of nutrition and influence development time, survivorship, and fertility [12–14]. In addition, live yeast accelerate larval development and feeding rate compared to heat-inactivated yeast or nutritional supplements, indicating a role for commensal yeast that extends beyond food [15]. However, most of the studies examining the functional role of yeast in *Drosophila* have been performed using commercial *Saccharomyces cerevisiae (*“Baker’s yeast”), a strain that is not associated with natural populations of flies [16–19]. As such, the relationship between host animals and their natural fungal symbionts as well as interkingdom interactions between fungal and bacterial members of the microbiome community are mostly unknown.

The Hawaiian *Drosophila* clade exhibit an intricate co-evolution and co-dependency with fungi and provide an exceptional opportunity for delineating the contribution of both bacterial and fungal kingdoms to host physiology. The subclade of picture wing *Drosophila* (PWDs) are endemic to the Hawaiian Islands and are notable for their relatively recent isolation and rapid speciation, with the majority of species having originated less than 3 mya [20]. As with other *Drosophila* groups, PWDs have a mutualistic relationship with yeast [21]. However, in contrast to continental drosophilids and lab-raised flies, Hawaiian PWD harbor a rich diversity of both fungi and bacteria in their gut despite having been raised for multiple generations in the lab (this study; [16]). A number of species are considered to be specialist feeders who appear to rely on particular sets of fungi, mostly from the genus *Saccharomyces*, as a source of their nutrition and means of identifying host plants [22, 23].

We used *Drosophila grimshawi*, a member of the PWD clade of flies, to dissect how bacteria, fungi, and their interactions modulate host physiology and microbiome stability. Our findings reveal that female, but not male, fecundity and mating drive are strongly affected by antifungal treatment, whereas antibacterial treatment suppresses male fecundity. Additionally, alterations in reproductive function are accompanied by sex-specific changes in cuticular lipid and fatty acid levels. The suppression of both microbial kingdoms by concurrent antibacterial and antifungal treatment results in an almost complete loss of fecundity for both females and males, an outcome that can be partly rescued through fecal microbiome transplants.

## Materials and methods

### Drosophila husbandry

The flies used for this study derive from an ~50 year old lab stock of *D. grimshawi*, a generalist species native to Maui Nui (recorded from Maui, Molokaʻi, Lanaʻi). Flies are reared on standard Wheeler-Clayton media [24]. Rearing and dietary details are provided in Supplementary Information.

### Antimicrobial treatment

Flies were separated by sex within 7 days of eclosion and raised on one of the following types of food for 21 d (time until sexual maturity): standard diet (control), standard diet supplemented with coconut oil (COil), antifungal treatment (AF), antibacterial treatment (AB), and antibacterial and antifungal treatment (AB+AF). The AB food consisted of standard media containing 50 μg/ml of ampicillin and kanamycin (VWR Life Science; both dissolved in water), 50 μg/ml of tetracycline (EMD Millipore Corp.; dissolved in 70% ethanol), and 15 μg/ml erythromycin (Acros Organics; dissolved in 100% ethanol). The AB concentrations are based on previous studies with *Drosophila melanogaster* [25–27]. The AF food contained 1.25 mM captan (N-trichloromethylmercapto-4-cyclohexene-l,2-dicarboximide; Sigma-Aldrich) with COil (300 μl/L standard media; Kirkland brand) to suspend the captan. Captan is a broad spectrum fungicide that inhibits ascomycete fungi, and epiphytic and wine yeasts including *S. cerevisiae* [28, 29]. Previous studies with *Drosophila* have found no evidence of toxic effects at the 1.25 mM dose [30, 31]. The AB+AF treatment included captan, COil, and each of the antibacterial drugs in the concentrations described above. The control oil (COil) food contained COil (300 μl/L). The antimicrobial drugs were mixed into food to ensure that flies ingested the drugs and to inhibit microbial growth in the food, the major source of microbes for the flies [22].

### Plating Drosophila tissue

Single flies (N = 6) were cold-anesthetized, rinsed twice in 2.5% bleach solution followed by phosphate buffered saline (pH 7.4; PBS), homogenized in 100 μl PBS, and serially diluted at 10^−1^ and 10^−2^. Ten μl of each dilution were plated onto YPD media with 50 μg/ml ampicillin, kanamycin, and tetracycline, and 15 μg/ml erythromycin (selective for fungal growth) or MRS with 0.38 mg/ml captan (selective for bacterial growth). Plates were incubated at 30°C for 2 days and microbial colony forming units (CFUs) from the 10^−2^ dilution were counted.

### High throughput amplicon sequencing and data analysis

Six or eight flies were prepared for each condition, with equal numbers of males and females. High throughput sequencing (HTS) was performed with MiSeq and 250 bp paired-end kits (Illumina, Inc., CA, USA). Post-processing of HTS data (read filtering, denoising, and merging) was performed using the “MetaFlow|mics” microbial 16S rRNA pipeline for bacteria and the Fungal ITS pipeline for fungi [32]. Details of DNA extraction, library preparation, data processing and analysis, and taxonomic assignment are provided described in Supplementary Information.

### Physiological assays

For all assays, unless otherwise stated, flies were treated for 3 weeks with antimicrobial or control treatment prior to testing. Detailed descriptions of the oviposition assays, ovary dissections, mating behavior procedures, lipid extraction, and analysis are provided in Supplementary Information.

Fecal transplants: The fecal transplant was generated by washing the sides of the vial with 200 μl sterile PBS (care was taken not to touch the surface of the food). The droplet was collected and divided into two aliquots. One aliquot was heat inactivated by placing the wash in an 80°C oven for 10 min. Virgin male and female flies were placed on AB+AF media for 7 days before switching to control food inoculated with 15 μl of active fecal wash or heat inactivated wash.

Lipid analysis and analysis by gas chromatography mass spectrometry (GCMS): For cuticular hydrocarbon extraction, three to five replicates were prepared per condition as previously described [33]. For fatty acid extraction, lipids were extracted and esterified for gas chromatography mass spectrometry analysis as previously described [34]. Analysis by GCMS was performed on a 7820A GC system equipped with a 5975 Mass Selective Detector (Agilent Technologies, Inc., Santa Clara, CA, USA) and a HP-5 ms column ((5%-Phenyl)-methylpolysiloxane, 30 m length, 250 μm ID, 0.25 μm film thickness; Agilent Technologies, Inc.). Instrument parameters and details of lipid analysis are provided in Supplementary Information.

Statistical analyses of physiological assays: A negative binomial regression was used to analyze count data found to be overdispersed (variance-to-mean ratio is greater than 1). For one-way ANOVAs and comparisons between two groups, data were first tested for normality using the Shapiro–Wilk test. A Kruskall-Wallis test with Dunn’s multiple comparison test or Mann–Whitney test was applied for non-normal data. Statistical tests, *p*-values and sample sizes are provided in the figure legends and figures. Analyses were performed using Prism (v. 10.1.2; GraphPad Software, Boston, MA) and R version 4.4.1 using the *glmmTMB* package.

## Results

### Manipulation of bacterial and fungal communities

**Figure 1:**
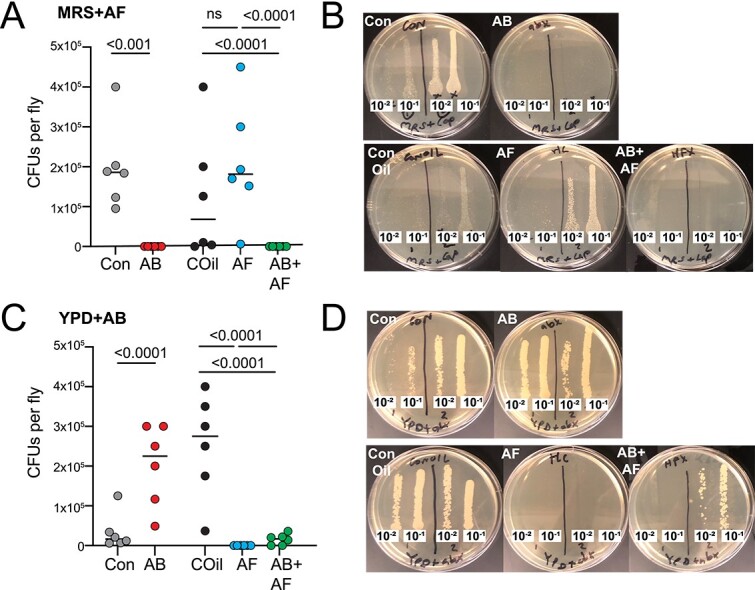
Efficacy of antimicrobial treatments. **A.** Antibacterial treatment (AB) eliminated almost all bacterial growth. Significantly fewer colony forming units (CFUs) appeared compared to controls (Con). Treatment with the antifungal captan (AF) led to a slight increase in bacterial CFUs relative to the respective COil-containing diet. The combined AB+AF treatment suppressed almost all bacterial growth. Individual circles represent the total CFU counts from single fly homogenate; line indicates median. The *p-*values were derived from a negative binomial model with N = 6 individual flies for each control and treatment group. **B.** Representative MRS + AF plates containing homogenate from single flies plated at two concentrations (10^−1^, 10^−2^), with two flies per plate. **C.** AF treatment suppressed fungal CFU growth compared to COil conditions. AB treatment led to an increase in fungal CFUs relative to the control diet. The AF and combined AB+AF treatments significantly reduced fungal growth. Individual circles represent CFU counts from single fly homogenate; line indicates median. The *p-*values were derived from a negative binomial model with N = 6 individual flies for each control and treatment group. **D.** Representative YPD + AB plates containing homogenate from single flies plated at two concentrations (10^−1^, 10^−2^), with two flies per plate.

To elucidate the separate contributions of bacterial and fungal microbiome components to host physiology, we first fed antimicrobial drugs to female and male *D. grimshawi* for 21 days to suppress the growth of bacteria, fungi, or both communities. *Drosophila* acquire their microbiome primarily from diet [35, 36]. Thus, the fly microbiome represents a mixture of gut and food communities [37]. Treating the media is expected to suppress microbial growth in the major source of microbes as well as within the gut. Homogenates of flies treated with antifungal or antibacterial drugs resulted in significantly fewer CFUs compared to control flies, indicating that 3 weeks of treatment were sufficient to significantly suppress microbial growth in the gut and that the antibacterial agents and captan are effective and selective inhibitors of *Drosophila* gut bacteria and fungi, respectively (Fig. 1; Tables S1, S2). The number of fungal CFUs increased with antibiotic treatment, indicating that in a healthy microbiome, the presence of bacteria may influence fungal growth.

### Changes in microbial community composition following antimicrobial treatment

**Figure 2:**
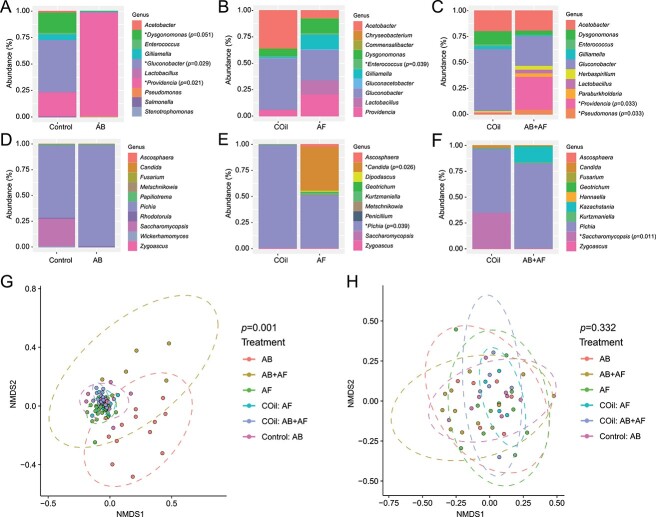
Microbiome profiles of control and experimental flies. **A–C.** Scaled relative abundance plots for the 10 most abundant bacterial genera following antibacterial (AB), antifungal (AF) or antibacterial and antifungal (AB+AF) treatment and the respective controls (Control or COil). Each column represents the average of 6–8 individuals based on high throughput 16S rRNA gene amplicon sequencing. The *p*-values were determined using univariate multiple testing with an F test; *: Relative abundance is significantly different between control and treatment conditions. **D–F.** Scaled relative abundance plots for the 10 most abundant fungal genera following AB, AF, or AB+AF treatment or the respective controls (con or COil). Each column represents the average of 6–8 individuals based on high throughput ITS gene amplicon sequencing. The *p*-values were determined using univariate multiple testing with an F test; *: Relative abundance is significantly different between control and treatment conditions. **G.** Non-multidimensional scaling plots (NMDS) based on Jaccard distances of bacterial OTUs (grouped at 97% similarity level) from control and experimental treatments. Ellipses represent significance at 0.05 confidence. **H.** Non-multidimensional scaling plots (NMDS) based on Jaccard distances of fungal OTUs (grouped at 97% similarity level) from control and experimental treatments. Ellipses represent significance at 0.05 confidence.

To determine how microbial community structure changes after treatment and whether each kingdom influences the others’ composition, we performed high throughput sequencing (HTS) of 16S rRNA and ITS amplicons from individual flies. *Acetobacter* and *Gluconobacter*, both of which are known commensals of wild *Drosophila* [38]*,* are the primary microbial components of both the fly gut and the dietary media (Fig. 2; Fig. S1A, C). Many control and treatment flies also hosted *Gilliamela*, a symbiotic bacterium found in honey bees [39]. Antibacterial (AB) treatment substantially reduced bacterial but not fungal taxonomic richness (Fig. S2A, D). In addition, both AB and combined antibacterial and antifungal (AB+AF) treatments resulted in a significant increase of *Providencia* (Fig. 2A, C). In contrast, AF treatment induced a slight but non-significant increase of bacterial and fungal diversity (Fig. 2B, E; Fig. S2B, E). Feeding flies both antibacterial and antifungal agents had little impact on 16S rRNA alpha diversity (Fig. S2C, F), indicating that the simultaneous suppression of both microbial kingdoms may have negated each kingdom’s impact on the overall community architecture. We note that the COil control groups exhibit different average relative abundance profiles compared to the other control group, particularly in levels of *Acetobacter*. Coconut oil is a source of medium chain fatty acids (MCFAs), a class of lipids that is known to alter microbiome composition in mammals [40]. Thus, the presence of MCFAs may contribute to the changes in microbiome composition.

With respect to the fungal profile, *Pichia* was the most common genus in both control and treated flies as well as the fly food (Fig. 2D-F; Fig. S1B, D). Captan altered the relative abundances of *Candida* and *Pichia* genera (Fig. 2E) while AB+AF treatment reduced *Saccharomycopsis* (Fig. 2F).

We next examined how microbiome community composition changes in response to each antimicrobial treatment. Bacterial composition was significantly different compared to the respective controls following AB, AF, or AB+AF treatment (Fig. 2G; Fig. S3A-C). In contrast, the composition of the mycobiome was not significantly altered by any of the drug treatments (Fig. 2H; Fig. S3D-F, H). The combined AB+AF treatment changed bacterial but not fungal composition in a manner that was distinct from either AB or AF treatment alone (16S rRNA: *p*-value 0.005; ITS: *p*-value 0.161; ANOSIM; Fig. S3G, H). Captan treatment resulted in sex-specific differences in microbiome composition (Fig. S4A, B), a shift that was mostly driven by a decrease of *Acetobacter* and *Lactobacillus* in females compared to males. In addition, the relative abundance of the yeast genera *Pichia* dropped precipitously in captan-treated females compared to males (Fig. S4C, D). There were no differences found between the mycobiome composition of males and females in control flies.

Taken together, manipulations of each microbial kingdom separately and together reveal that the fungal community is more resilient to compositional changes, whereas bacterial community stability appears to be partly dependent on the composition of the fungal microbiome. Our findings also indicate that males and females respond differently to antifungal treatment although sex-specific patterns of feeding behavior may also play a role (see Discussion).

### Bacterial and fungal dysbiosis have different effects on host reproduction

After confirming the efficacy and specificity of the antimicrobial treatments, we next characterized the impact of bacterial or fungal dysbiosis on reproduction and related physiological features.

#### Fecundity

**Figure 3:**
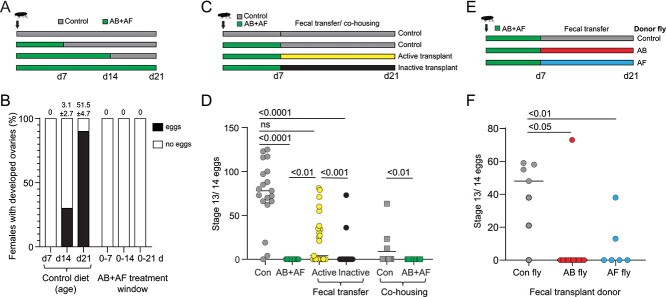
The role of the microbiome in ovary development. **A.** Feeding scheme for antimicrobial treatment. Newly eclosed flies were continuously fed antibacterial and antifungal (AB+AF) treatment for 7, 14, or 21 d. **B.** Percentage of *Drosophila grimshawi* with developed ovaries, defined as the presence of stage 13/ 14 mature eggs. The majority of females fully develop ovaries between 2–3 weeks post-eclosion. Suppressing the microbiome during the first 3 weeks post-eclosion completely inhibits oogenesis. The average mature egg number (± standard error of mean) is indicated at the top of each bar; N = 10 per condition. **C.** Feeding scheme used for antimicrobial treatment and fecal transplant. Flies are fed AB+AF treatment for 7 days then switched to control media supplemented with active fecal transplant or heat-inactivated fecal transplant for 14 days. Rescues were also performed by co-housing treated flies with control flies or AB+AF treated flies. **D.** The AB+AF treatment suppressed stage 13/14 egg production (con: N = 19; AB+AF: N = 10). Fecal transfers from control flies (N = 25) or co-housing with control flies (N = 11) partially rescued oogenesis compared to heat-inactivated fecal transfer (N = 28) or co-housing with AB+AF flies (N = 11). Individual points represent single flies; line indicates median. The *p-*values were derived from a negative binomial model. **E.** Feeding scheme for fecal transfers from AB- or AF-treated donors. Flies are fed AB+AF treatment for 7 days then switched to control media supplemented with active fecal transplant from AB- or AF-treated donors. **F**. Fecal transfers from control flies (N = 7) rescued oogenesis whereas fecal treatments from AB- (N = 11) or AF-treated donors (N = 6) failed to restore egg development. The *p-*values were derived from a negative binomial model with N = 6 individual flies for each control and treatment group.

Previous studies of gnotobiotic and axenic flies established that gut bacteria support host fecundity and fertility [41–43]. To determine whether *D. grimshawi* exhibit a similar dependency on their microbiome, we first measured fecundity in flies after treatment with both antibacterial and antifungal drugs. Normally, lab-raised *D. grimshawi* females begin to develop ovaries between 7–14 days post-eclosion and reach full sexual maturity with ovaries containing Stage 13/14 eggs between 14–21 days post-eclosion (this study; [44]) (Fig. 3). When the fly microbiome is suppressed during the first week of adult development, no mature eggs develop (Fig. 3A, B). To address the possibility that off-target effects of the antimicrobial treatments inhibit oogenesis, we tested whether restoration of the microbiome via frass transplant or co-housing with control flies could rescue egg production. Of the flies inoculated with active fecal transfer from control flies, 52% developed mature eggs, compared to only 7% of females treated with heat-inactivated fecal transfer (Fig. 3C, D; Table S3). Additionally, co-housing AB+AF-treated flies with a control fly resulted in 25% of females with mature ovaries. By comparison, female ovaries remained undeveloped when co-housed with another AB+AF female (Fig. 3D; Table S4).

Lastly, active fecal transplants harvested from AB- or AF-treated flies were substantially less effective in restoring fecundity than rescue with fecal transplants from control flies: 86% of females inoculated with feces from control flies developed mature eggs, compared with 9% or 33%, respectively, of females inoculated with feces from AB- or AF-treated flies (*p*-value 0.003, Fisher exact probability test, N = 6–11; Fig. 3E, F; Table S5).

To determine whether rescue of oogenesis is associated with changes in the microbiome profile, we performed HTS amplicon analysis of flies from each of the inoculation conditions and found significant differences in diversity and composition. Flies receiving active frass transplants from control flies exhibited a different microbiome composition compared to flies inoculated with PBS or inactivated frass (for both, **p-*value* 0.003; ANOSIM with post-hoc pairwise PERMANOVA, N = 9–12; Fig. S5A-C). In particular, active frass transplants restored *Gluconobacter* and *Providencia* levels similar to those of control animals (for both taxa, *p*-value 0.5602; ANOVA with post-hoc Tukey’s multiple comparisons test), although the overall bacterial community composition remained different (*p*-value 0.003, ANOSIM with post-hoc pairwise PERMANOVA). In contrast to the impact on bacterial composition, the fungal composition was not altered by any of the transplant treatments (**p-*value* 0.492; ANOSIM; Fig. S5D-F).

Taken together, these results reveal that antimicrobial treatment during a critical developmental window in early adulthood profoundly alters ovary development. Microbial activity may supply oogenesis-promoting metabolites and the microbes themselves may serve as a source of nutrition. Secondary effects from the antimicrobial drugs may also contribute to the loss of fecundity. However, the partial restoration of fecundity from frass transplantation shows that microbes can partially compensate for dysbiosis or drug-related toxic effects.

**Figure 4:**
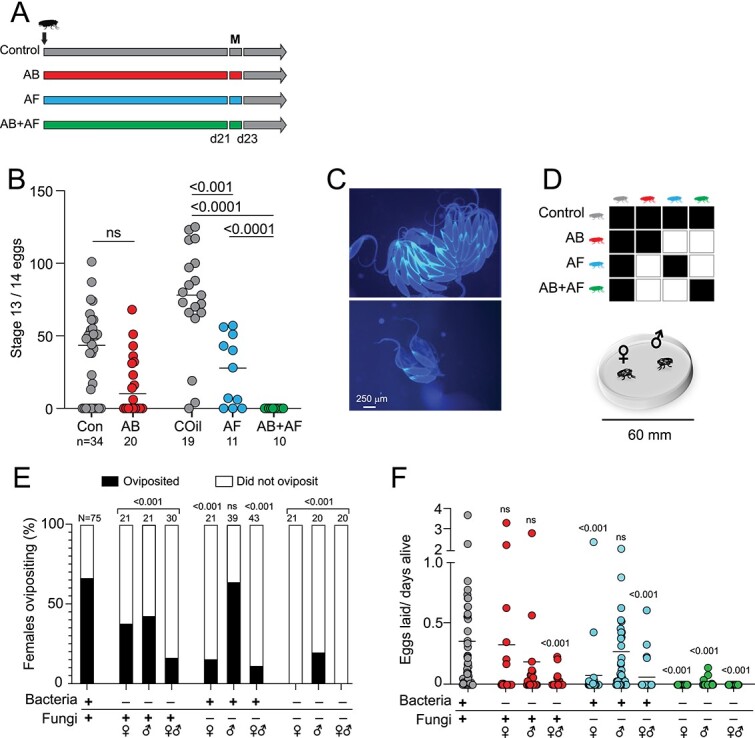
Distinct effects of antimicrobial treatments in male and female fecundity. **A.** Feeding scheme to test the role of bacteria and fungi in oogenesis. Newly eclosed flies are fed antibacterial (AB), antifungal (AF), or AB+AF treatments for 21 days, mated for 2 days (M), then maintained on control food for oviposition assays. **B.** AB treatment of virgin females causes a slight decrease in the number of mature stage 13/14 eggs compared to control flies. Females treated with AF or AB+AF produce significantly fewer eggs compared to the respective control. Treatment with AB+AF suppressed oogenesis to a greater extent than either drug alone; lines indicate median. The *p-*values were derived from a negative binomial model with N = 6 individual flies for each control and treatment group. Samples sizes are indicated beneath each treatment. **C.** Representative images of ovaries from flies fed on control oil food (top) or captan-supplemented food (bottom). **D**. Mating combinations used to test the role of bacteria and fungi in male and female fecundity. One male and one female from control, antibacterial (AB), antifungal (AF), or AB+AF treatments are placed in each courtship chamber. Flies are monitored for 48 h. **E**. AB treatment of females, males, or both sexes led to reduced fecundity. Significantly fewer AF-treated females oviposited but AF treatment had no substantial impact on male fecundity (*p*-value 0.53). Concurrent treatment with AB and AF significantly reduced male and female fecundity. Samples sizes are indicated above bars. The *p*-values were determined by a two-tailed fisher exact probability test. **F**. AB treatment of females or males did not substantially change the number of eggs laid (N = 21; *p*-value 0.14). However, AB female/ male dyads exhibit an additive loss of fecundity (N = 30; *p*-value < 0.0001). AF-treatment of females only significantly inhibited the number of eggs laid (N = 38–39; AF females: *p*-value < 0.0001). AF treatment of both males and females inhibits fecundity (N = 43; *p*-value < 0.0001)). AB+AF treatment results in the loss of fecundity in both females and males compared to controls (N = 20; AB+AF males: **p*-value =* 0.0002; AB+AF females and AB+AF dyads: *p*-value < 0.0001). Lines indicate median. The *p*-values were determined by a Kruskal-Wallis test with Dunn’s multiple comparisons test.

To assess the relative roles of bacterial and fungal activity in fecundity, we first selectively suppressed each kingdom by adding antimicrobial drugs to the food and measured ovary development (Fig. 4A-C; Table S6). Virgin females treated with antibacterial drugs developed fewer mature eggs compared to the control group (Con: 35.3 ± 5.1 vs AB: 19.6 ± 5.3; mean ± SEM). Antifungal treatment suppressed oogenesis to a greater degree compared to the control oil (COil) group (COil: 75.8 ± 8.3 vs AF: 26.2 ± 7.2). However, concurrent manipulation of bacteria and fungi resulted in minimally developed ovaries with significantly fewer mature eggs compared to inhibiting either antimicrobial treatment alone, indicating potential interactions between the kingdoms (AB vs. AB+AF: *p-*value < 0.0001; AF vs AB+AF: *p*-value < 0.0001; negative binomial regression).

Next, we measured male and female fecundity in the context of mating. Female oogenesis and oviposition behavior are enhanced by mating due to the transfer of sex peptide [45] and accessory gland proteins from males [46, 47]. As such, the egg laying frequency and the number of eggs laid provide measures of both female and male fecundity. We placed single males and females in a mating chamber and measured the percentage of females that oviposited (an indicator of successful copulation) and the number of eggs laid following microbiome manipulation of males, females, or both sexes (Fig. 4D). Bacterial dysbiosis in females or males led to a significant decrease in the percentage of females that oviposited as well as males’ ability to induce oviposition (Fig. 4E). However, the number of eggs laid by AB-treated females did not change compared to controls (Fig. 4F). In contrast, antibacterial treatment of both members of the mating pair substantially decreased the number of eggs laid (Fig. 4F).

Application of antifungal treatment or both antibacterial and antifungal treatments significantly reduced egg laying: only 15.8% of captan-fed females oviposited (Fig. 4E, F). While treatment with AB+AF fully inhibited oviposition, the effect was not significantly different from AF treatment alone (*p*-value 0.12, one tailed Fisher exact probability test). Similarly, the effect of AB+AF on the number of eggs laid was comparable to AF treatment alone (*p*-value 0.44, Kruskal-Wallis with Dunn’s multiple comparison). Both outcomes indicate that the impact on egg-laying observed in response to AB+AF treatment is largely due to the AF effects.

Male fecundity exhibited a different response to microbiome manipulation compared to females, and was more affected by AB treatment compared to AF treatment. We assessed male fecundity based on the ability to induce control females to oviposit, an indicator of successful copulation [48]. As with females, bacterial dysbiosis had a negative impact on males’ ability to induce egg laying (Fig. 4E, F). However, unlike females, fungal dysbiosis had little effect on male fecundity as indicated by two measures: the high proportion of partnered control females that oviposited and the number of eggs laid, both of which were indistinguishable from controls. With AB+AF treatment, male fecundity was profoundly dampened (Fig. 4E, F). Only 20% of control females paired with AB+AF males oviposited. Of the ones that successfully mated, the egg laying rate was reduced to 0.01 eggs/day, compared with 0.4 eggs/day for controls. The impact on oviposition and egg laying caused by AB+AF dysbiosis was similar to that caused by AB alone (oviposition: *p*-value 0.11, one-tailed Fisher exact probability test; eggs laid: *p*-value 0.35, Kruskal-Wallis with Dunn’s multiple comparison). In summary, males and females respond differently to antibacterial and antifungal treatments: the former has a greater impact on male fecundity whilst the latter suppressed oogenesis and egg laying. Concurrent administration of both antimicrobial drugs fully inhibited ovary development, implicating a role of bacterial-fungal interactions in oogenesis.

#### Mating

**Figure 5:**
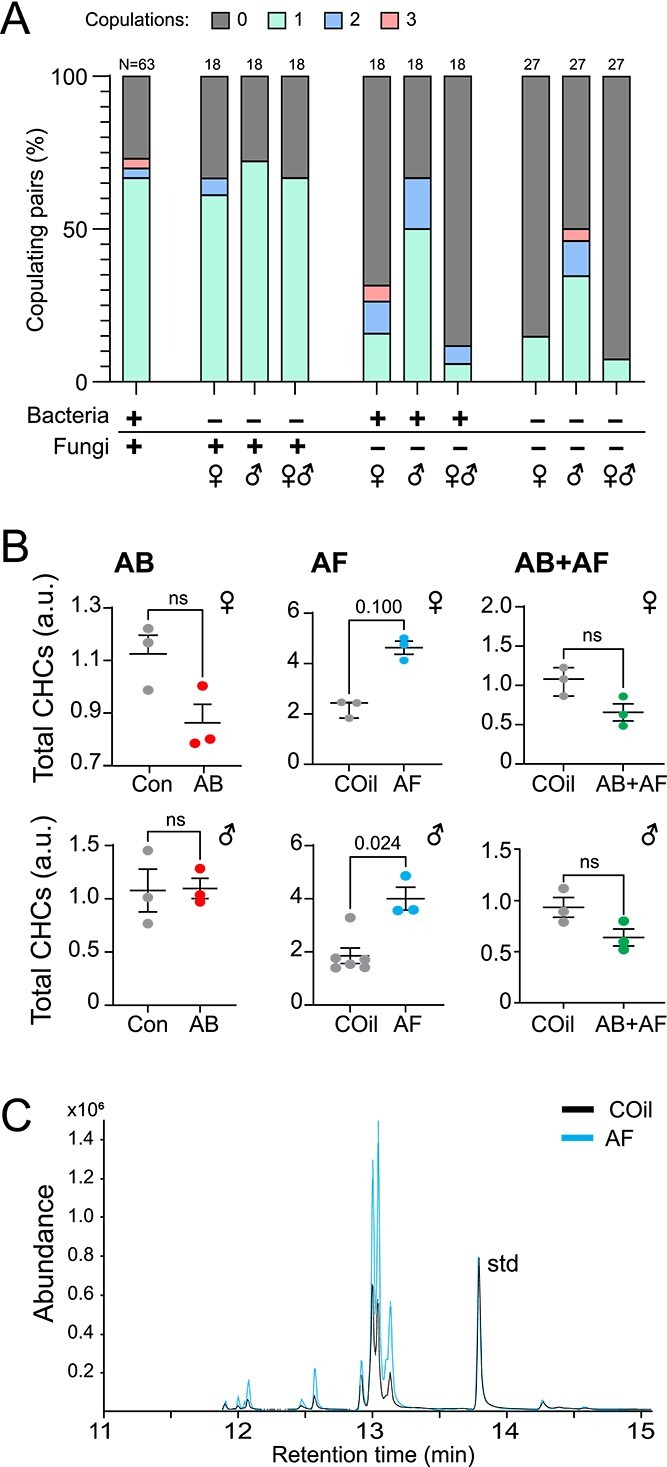
Impact of antimicrobial treatment on mating and cuticular hydrocarbons. **A**. Proportion of females copulating 0, 1, 2, or 3 times during 48 h mating trial. Copulation occurred with similar frequency amongst AB-treated flies. AF-treated females, but not males, were significantly less likely to mate (*p*-value 0.001). Mating amongst pairs with both AF-treated males and females was also significantly suppressed (*p*-value < 0.0001). Dyads involving AB+AF females copulated with lower frequency (*p*-value < 0.0001) but AB+AF males had only a slight decrease in on mating frequency (*p*-value 0.049). Multiple copulations were more frequent in trials involving AF treated flies and in particular, AF males. Samples size is indicated above bars. The *p*-values were determined by a fisher exact probability test. **B**. Cuticular hydrocarbon (CHC) total abundances. Overall levels of CHCs are higher in AF-treated females and males (N = 3–6; Mann Whitney test). The CHC levels of AB and AB+AF-treated flies were unchanged by antimicrobial treatment; lines indicate mean ± S.E.M. each replicate consists of extract from three flies. **C**. Representative GCMS chromatogram overlaying the CHC profile from an AF-treated female with a control showing a notable difference in signal abundance for all major peaks. The chromatograms are normalized to a spiked standard (std).

Our observations that AF and AF + AB treatments resulted in females laying fewer eggs can be partly explained by a loss of fecundity. However, it may also be the case that fewer of the treated flies copulated. To address whether the microbiome influences mating decisions, we counted the frequency of copulation events in paired males and females (Fig. 5A). Control dyads mated 73% of the time. Both males and females on AB diets exhibited little change in mating frequency compared to control flies. However, significantly fewer females laid eggs despite mating (Fig. 4E; *p*-value < 0.0001, Fisher exact probability test), indicating that AB treatments reduced the likelihood of successful copulation.

In contrast to the limited effect of antibiotic treatment, antifungal treatment had a considerably greater impact on female, but not male, mating drive. Significantly fewer females treated with antifungals (either alone or in combination with antibiotics) copulated compared with control females, regardless of the male treatment group (Fig. 5A; AF: *p*-value 0.012; AB+AF: *p*-value < 0.0001, Fisher exact probability test). In contrast, the frequency of male mating and of successful copulation did not change with captan treatment. Fungal suppression in males either with AF or AB+AF treatments tended to increase instances of multiple copulations. Taken together, the mating behaviors of females and males were differentially affected by microbiome manipulation: female mating drive and successful copulation are influenced by both antimicrobial treatments whereas male mating drive is largely unaffected.

One important pre-copulatory trait that robustly influences the decision to court is pheromone signaling. For many insects, cuticular lipids function as sex pheromones that impel or inhibit the decision to mate. Antifungal treatment led to a significant increase of both male and female cuticular hydrocarbon (CHC) levels (Fig. 5B, C; Tables S7, S8). The observed difference in cuticular lipids indicates that in females, the microbiome has a systemic impact on multiple reproductive features of reproduction including ovary development and cuticular lipid levels, with the latter potentially serving as an honest indicator of fitness.

#### Fatty acid profiles

**Figure 6:**
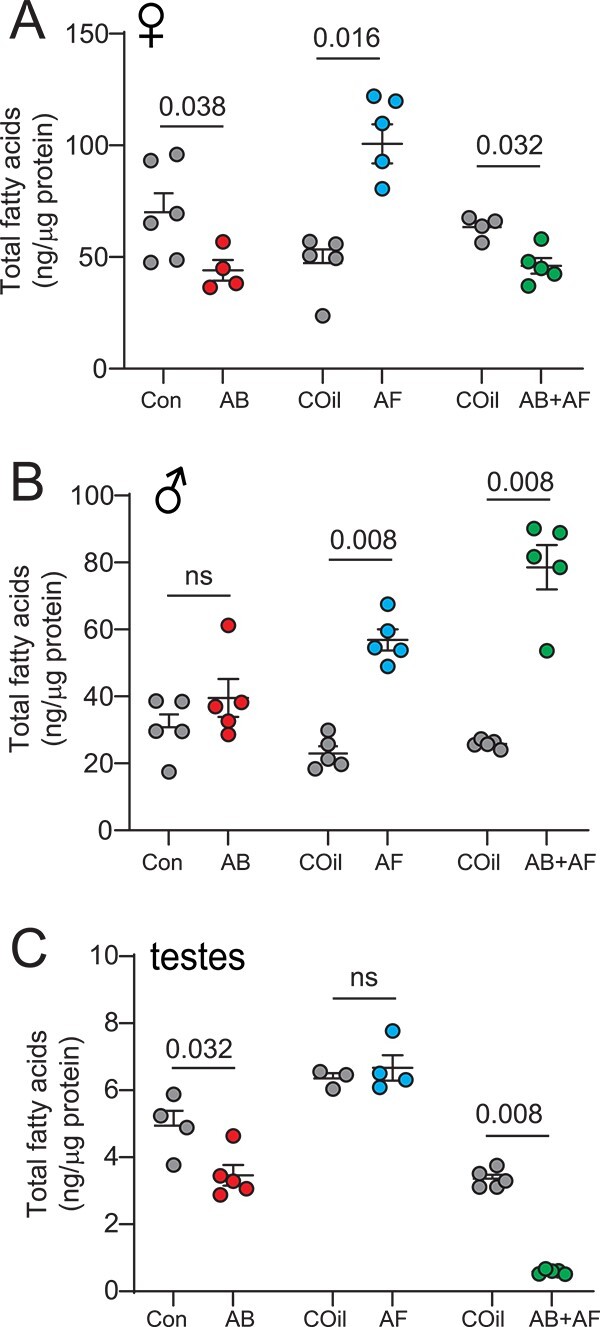
Impact of antimicrobial treatment on the fatty acid content of the whole body and testes. **A.** Females treated with AB had lower total fatty acid (FA) levels compared to controls (Mann Whitney test). AF-treated females contained significantly more total FAs compared to respective controls. AB+AF treatment resulted in decreased FA levels; N = 4–6 for all treatments; mean ± S.E.M. are shown. Each replicate consists of extract from three flies. **B.** Males treated with AB had similar total FA levels compared to controls (Mann Whitney test). Fatty acid content increased significantly with AF or AB+AF-treatment; N = 5–6 for all treatments, mean ± S.E.M. are shown. Each replicate consists of extract from three flies. **C**. Testes of AB-treated males contained significantly lower FA levels (Mann–Whitney test). Unlike the results from whole body samples, FA levels in the testes were unchanged by AF treatment. The FA levels dropped significantly under AB+AF conditions; N = 4–6 for all treatments, mean ± S.E.M. are shown. Each replicate consists of extract from three flies.

Reproduction is an expensive physiological process that requires a substantial investment of energy stores and can result in the diminution of life span for females [49]. Given the robust effects of microbiome dysbiosis on male and female fecundity, we next sought to determine whether whole body fatty acids (FA), a major storage source of energy, are also affected. Antibacterial treatments, which slightly reduced oogenesis, lowered fatty acid levels in females but not males (Fig. 6A; Table S9). In contrast, AF and AB+AF treatments, both of which had sizeable effects on female reproduction, caused significant changes in total FA levels, although in opposite directions (Fig. 6A; Table S9), and led to a shortening of fatty acid carbon chain length (Fig. S6). The most striking effects occurred with captan treatment which induced a near 2.5-fold increase in FAs. The combined antimicrobial treatment, which eliminates almost all egg production and egg laying, resulted in a substantial decrease of FAs, a finding that contrasts with the outcomes of antifungal treatment alone *(*Fig. 6A; Table S9; *p*-value 0.039, Kruskal-Wallis with Dunn’s multiple comparison)*.* As with fecundity, co-treatment with both antimicrobial drugs impacted lipogenesis in a manner that is distinct from either drug alone.

For males, no significant differences in whole body FA levels were found with AB treatment (Fig. 6B; Table S10). The FA levels increased with captan administration, as seen with females (Fig. 6B; Table S10). In addition, the FA species shifted towards shorter carbon chain lengths for all treatments (Fig. S7A-C). However, in contrast to females, male FA levels increased substantially when both antimicrobials are provided compared to AB but not AF treatment (AB vs. AB+AF: *p*-value 0.09; AF vs AB+AF: *p*-value 0.31; Kruskal-Wallis with Dunn’s multiple comparison; Fig. 6B; Table S10). We next asked whether the microbiome dysbiosis influences lipid metabolism in the testes. Fatty acids serve as useful indicators of reproductive health since they function as key components of phospholipids in the sperm cell membrane [50–53] and are needed for germ cell maturation [54, 55]. Consistent with our findings that AB and AB+AF-treated flies exhibit reduced fecundity, FA levels in the testes decreased in response to either treatment (Fig. 6C; Table S11) and the effect was stronger with the combined treatment (*p*-value 0.0074, Kruskal-Wallis with Dunn’s multiple comparison). The outcomes possibly indicate a reduction in metabolic activity or lower levels of sperm production. In addition, FAs shortened in response to AB+AF treatment (Fig. S7F) and unsaturation levels increased in response to AB treatment (Fig. S7J). FA levels and profiles in the testes did not change with AF administration (Fig. 6C; Fig. S7E, K; Table S11). Overall, treatments that induced fecundity loss in males are associated with a reduction in testes FA levels. Moreover, the changes in testicular FA profiles are distinct from those observed in the whole body following antimicrobial treatment.

## Discussion

By using antimicrobial drugs to separately and concurrently manipulate bacterial and fungal communities associated with Hawaiian *Drosophila*, we identified distinct effects of each kingdom and their interactions on male and female fecundity, mating behavior, and lipid metabolism. Because our housing paradigm allows for the continuous exchange and intermixing of gut microbes and food microbes, the phenotypic effects we observed are the result of interactions between the fly microbiome, antimicrobial drugs, food, frass, and the associated metabolic products. In contrast to generating axenic animals, a pharmaceutical-based strategy allows microbes to be conditionally suppressed only at the post-eclosion stage, thus avoiding developmental effects. There are, however, some drawbacks to using antimicrobials. First, captan does not inhibit all fungal types and may also suppress some bacterial taxa [56, 57]. Although plating on MRS and YPD media showed that each antimicrobial treatment effectively suppressed each targeted kingdom, microbial growth on media represents only a subset of the total gut microbiome. As such, the actual amounts of active microbes in both control and treated flies are likely to be higher than indicated by CFU counts. A second drawback is that prolonged exposure to antimicrobial drugs may have direct effects on host cellular processes and physiology [58, 59]. While we cannot exclude this possibility, two lines of evidence suggest that the phenotypes observed are due to microbiome dysbiosis and suppression rather than secondary effects: first, previous work examining the impact of captan on *D. melanogaster* found no evidence of toxicity when used in the range of 10–100 mM [31] or 10–50 mM [30], a dosage range that is an order of magnitude higher than the amount used in our studies. Second, oogenesis defects were partially rescued by active, but not inactivated, frass transplants from control flies, indicating that microbial activity supports fecundity and is able to compensate for acute drug effects. Notably, the microbiome composition was not fully restored, consistent with our observations that female fecundity was only partially rescued. The bacterial profiles of flies inoculated with active frass revealed some overlap with control fly microbiomes—*Gluconobacter* and *Providencia* were restored to levels similar to those found in control flies. Examining the role of these taxa in reproduction will help to elucidate molecular factors and metabolites involved in microbe-host interactions. Transplanted microbes may also benefit the host by serving as a source of nutrition, as has been observed for *D. melanogaster* [60–62]. Active microbes will have greater biomass and hence, nutrient density compared to inactivated microbe transplants, a factor that was not controlled for [60]. We note, however, that active transplants from AB- or AF-treated flies were less effective at rescuing fecundity.

### Bacterial and fungal communities exhibit different compositional stability

Overall, our manipulations of the *Drosophila* bacterial-fungal microbial community reveal that whilst bacteria composition changes with both antibacterial and antifungal administration, fungi composition is comparatively stable. No significant changes in fungal beta diversity were detected under any of the treatment conditions (although the relative abundance of *Saccharomycopsis* fungi increased with antibacterial treatments). In contrast, the relative abundance of *Enterococcus* populations increased by nearly 2-fold with fungicide administration. Similar outcomes have been observed in mammals whereby the administration of antifungal drugs in a model of mouse colitis resulted in an expansion of bacterial genera [63]. The changes to the bacterial community could be due to interactions with the drugs, changes in the relative abundance of fungi relative to bacteria, or changes in select bacterial taxa allowing other microbes to proliferate.

### Impact of the microbiome on female and male fecundity

Previous work has already established that *D. melanogaster* exhibit sex differences in immune and metabolic responses to infection and microbiome composition, respectively [64, 65]. Here, we show that female reproduction and microbiome composition is more strongly affected by antifungal treatment compared to males (Fig. 4, 5). In particular, captan treatment led to a reduction of *Acetobacter* and *Lactobacillus* in females but not males. Both taxa have been linked to the fertility of *D. melanogaster* [41, 42, 66]. We note, however, that antibacterial treatment, which also reduces the relative abundances of *Acetobacter* and *Lactobacillus (*Fig. 2), had only slight effects on oogenesis, indicating that captan-induced changes in the bacterial community are not the primary cause of female fecundity loss. The male–female difference in captan response may be due to sexually dimorphic differences in feeding preference and food intake [67–70]. Careful quantitation of drug intake would help to address the potential confound.

In addition to a loss of fecundity, female mating drive was also reduced upon antimicrobial treatment. This behavioral shift may either be due to an increased reluctance to mate or a decreased attractiveness to males. Limiting mating activity in the absence of ovary development or a compromised immune defense system may be a reproductive strategy to conserve resources, reduce the risk of infection, and minimize physical damage [71–73]. Microbes also play sexually dimorphic roles in foraging and locomotion [74, 75]. Alterations in motor behavior may also contribute to the change in female mating drive. Although female CHCs levels increased with captan treatment, it is unclear whether males perceive and respond differently to the change. In other insect species, total CHC levels increase with age [75–77]. Indeed, *Drosophila melanogaster* use features of the CHC profile as an indicator of the age of the potential mate [78].

### How do microbes influence fecundity?

Our fecundity rescue experiments reveal that only active fecal transfers from control donors are capable of partially restoring female fecundity and this partial rescue is associated with an increase in the relative abundance of *Gluconobacter* and *Providencia* (Fig. 3D, F). Neither co-housing with AB+AF- treated flies nor fecal transfers from AB- or AF-only flies were effective in fully restoring fecundity although microbiomes from AF-treated flies rescued oogenesis in two of six flies. Considering that AB- or AF-treated flies have higher abundances of, respectively, fungi or bacteria based on CFU counts (Fig. 1), these outcomes indicate that microbial biomass by itself is not sufficient to reverse the deleterious effects of microbiome suppression; rather, microbe composition also plays a role in supporting oogenesis, consistent with previous studies in *D. melanogaster* [41, 43]. Because lipid metabolism and reproduction are mechanistically coupled through numerous shared metabolic underpinnings [79], the microbiome could influence fecundity by modulating FA composition. Previous studies have shown that microbes supply FA precursors and lipid profiles change in response to the elimination of bacteria [80] or a shift in fungal and bacterial composition [65]. Although AB+AF treatment was detrimental for both male and female fecundity and mating (Fig. 4, 5), the overall FA levels changed in opposite directions for each sex. For females, changes in FA levels that result from shifts in the microbial community likely influence the balance between fat storage and reproduction. As for male fecundity, we observed that FA levels in the testes dropped significantly with the suppression of bacteria or both bacteria and fungi and, respectively, patterns of saturation and carbon chain length were altered. Shifts in FA length can alter functional properties of spermatozoa including membrane fluidity and sperm motility [53] and may underlie the loss of fecundity. Alternatively, energy stores could be shunted from spermatogenesis or accessory gland protein production to support other physiological needs such as immune defense [80–83], telomere length (indicator of aging and lifespan) [84], and defensive weapons [85]. It is also possible that the antimicrobials used in this study have direct effects on lipid production and male fertility. The antibiotic tetracycline suppresses male but not female fertility in *D. melanogaster* when used at a 250 μg/ml dose (5 times higher than the dose used in our experiments [59]). Disentangling the direct effects of the antimicrobial drugs from the microbe-related impact will be explored in the future with the use of axenic *D. grimshawi*. Overall, our findings reveal that the microbiome, and in particular, bacterial-fungal interactions modulate fecundity and fatty acid levels throughout the whole body as well as in the testes and have differential impact on males vs. females.

### Implications for the rapid radiation of Hawaiian Drosophila

*Drosophila*, like many animals, rely on external sources such as diet to renew their microbiome [35, 38, 86]. The diversity of yeast associated with Hawaiian PWDs and their host plants has been proposed as a factor in PWD rapid speciation. Given our findings that the mycobiome is coupled to female fecundity and mating drive, variation in fungal communities either through host plant selection or abiotic conditions may contribute to reproductive isolation. In addition to fecundity, our results reveal that CHC levels are also influenced by microbe composition. Considering that CHCs serve as a barrier to desiccation, these outcomes have fascinating implications for the role of microbes in enabling host tolerance to local temperature and humidity conditions. As such, microbes acquired from host-plants may have contributed to PWDs explosive radiation across the Hawaiian archipelago by facilitating rapid adaptation. Future experiments measuring the interaction between microbe composition and temperature and humidity tolerance under natural conditions are needed to address this prediction.

## Supplementary Material

Supplemental_text_final_ycae134

## Data Availability

High throughput sequencing data have been deposited with links to BioProject ac- cession number PRJNA1176301 in the NCBI BioProject database (https://www.ncbi.nlm.nih.gov/bioproject/). The datasets for physiological assays are available at Zenodo for download (https://doi.org/10.5281/zenodo.13956531). The authors declare no competing financial interests in relation to the work described.
