# Supplementary material for: Bacterial and fungal components of the microbiome have distinct roles in Hawaiian Drosophila reproduction
Source: ISME Commun. 2024 Nov 4;4(1):ycae134. doi: 10.1093/ismeco/ycae134 (PMC11643357; doi:10.1093/ismeco/ycae134)
Supplement: Supplemental_text_final_ycae134 [file supplemental_text_final_ycae134.pdf]

Bacterial and fungal components of the gut microbiome have distinct roles in Hawaiian *Drosophila* reproduction

Matthew J. Medeiros[1,2], Laura Seo[2], Aziel Macias[2], Donald K. Price[2], and Joanne Y. Yew[1,2*]

[1] Pacific Biosciences Research Center, University of Hawaiʻi at Mānoa
[2] Department of Life Sciences, University of Nevada at Las Vegas

*corresponding author: jyew@hawaii.edu

Supplementary Materials and Methods

Drosophila husbandry and diet

Fly cultures and all experimental treatments and assays were maintained in an incubator held at 18 °C, 60% relative humidity with a 12 hr/ 12 hr day/ night cycle. Flies were reared on a standard diet Wheeler-Clayton diet (Carson 1987) consisting of 90 mL water, 1 g agar, 24 g Gerber baby banana food, 3 g powder mix (made by blending equal parts wheat germ, textured soy protein, and Kellog's Special K cereal), 375 µL proprionic acid (Avantor; Radnor, PA), and 375 µL 100% non-denatured ethanol (Decon Labs Inc., King of Prussia, PA).

High throughput sequencing

Flies were surface sterilized with 2 washes in 95% EtOH followed by 2 washes in sterile water. Six to eight flies were prepared for each condition, with equal numbers of males and females. Individual flies were homogenized in ATL buffer from PowerMag Bead Solution (Qiagen) with 1.4 mm ceramic beads (Qiagen; MD, USA) using a bead mill homogenizer (Bead Ruptor Elite, Omni, Inc; GA, USA) and extended vortexing for 45 min at 4 °C. The homogenate was treated overnight with proteinase K (2 mg/ mL) at 56 ˚C and DNA was extracted using the MagAttract PowerSoil DNA EP Kit (Qiagen) according to manufacturer's instructions. Bacterial diversity was characterized by PCR amplification of the 16S rRNA gene with primers to the V3-V4 region (515F: GTGYCAGCMGCCGCGGTAA; 806R: GGACTACNVGGGTWTCTAAT) (Parada et al 2016). Fungal diversity was characterized using primers to the internal transcribed spacer (ITS1f: CTTGGTCATTTAGAGGAAGTAA; ITS2: GCTGCGTTCTTCATCGATGC) (White et al 1990). The primers contain a 12-base pair Golay-indexed code for demultiplexing.

PCRs were performed with the KAPA3G Plant kit (Sigma Aldrich, MO, USA) using the following conditions: 95 °C for 3 min, followed by 35 cycles of 95 °C for 20 seconds, 50 °C for 15 seconds, 72 °C for 30 seconds, and a final extension for 72 °C for 3 min. The PCR products were cleaned and normalized with the Just-a-plate kit (Charm Biotech, MO, USA). High throughput sequencing (HTS) was performed with Illumina MiSeq and 250 bp paired-end kits (Illumina, Inc., CA, USA).

High throughput sequencing data analysis

Reads shorter than 20 bp and samples with fewer than 5,000 reads were discarded. Paired reads are merged if the overlap is at least 20 bp with a maximum 1 bp mismatch. The contigs generated by DADA2 (Callahan et al 2016) were processed using MOTHUR (Schloss et al 2009) and initially aligned and annotated using the SILVA v138 database (Quast et al 2013). We chose a 97% sequence similarity cutoff for determination of OTUs as we were attempting to confirm the efficacy of the antimicrobial drugs rather than investigate specific strains of microbes. Sequences that were unassigned by the pipeline were identified by manual searches in NCBI BLAST, UNITE (Nilsson et al 2019), and MycoBank (Robert et al 2013) using a >95% sequence similarity cutoff. The ITS data were normalized in R using the DESeq2 package (Love et al 2014). Analyses were performed after clustering at the genus level using R version 4.2.1, and the phyloseq package (McMurdie and Holmes 2013).

Oviposition

Following 3 weeks of antimicrobial or control treatment, a single male and single female were placed in a standard 8-dram fresh control food vial for 48 hrs, after which males were removed.

Food for egg laying trials contained one drop of blue food coloring (Spice Supreme, Bayonne, NJ) per batch to increase the visibility of eggs. Females were transferred to new food vials twice per week and outgoing food vials were checked for eggs for the lifespan of the female. Vials containing eggs were checked for larvae twice a week.

Ovary dissections

Flies were anesthetized on ice and ovaries were dissected in PBS after 21 days of treatment. Mature eggs were manually counted under 10x magnification. For ovaries used in images, the tissue was fixed in 4% paraformaldehyde for 20 min. and washed thrice with PBS + 0.1% TritonX-100 (PBST). Ovaries were dyed with Hoescht dye (1 µg/ mL) for 10 min, washed in PBS for 10 min., and mounted on slides with SlowFade Diamond Antifade mountant (ThermoFisher Scientific, Waltham, MA). Images were obtained by epifluorescence microscopy on an Olympus BX51 microscope equipped with a Leica DFC 7000 T color digital camera.

Mating behavior

A single virgin male and female were placed in a polystyrene Petri dish (60 x 15 mm) containing 0.5 mL of control food and a small piece of moistened filter paper and monitored for 48 hr. Each trial consisted of 36 dishes with 9 replicates of each pairwise mating combination: control male + control female, control male + treatment female, treatment male + control female, and treatment male + treatment female.

Mating behavior was monitored with Raspberry Pi computers outfitted with a camera (CanaKit Raspberry Pi 4 8GB computers with Longruner 1080p HD Webcam 5MP OV5647 IR-Cut Video cameras), with the location of Petri dishes randomized. Images of the flies were taken every 60 or 90 s for a duration of 48 h and subsequently analyzed manually for copulation events, defined as the male positioned directly behind the female with both female wings expanded.

Cuticular hydrocarbon extraction

Flies were cold anesthetized at 4 °C, placed in glass vials, and covered with 240 µL of hexane spiked with 10 ug/ mL hexacosane for 10 min at RT. Next, 200 µL of solvent was removed, added to a fresh vial and evaporated under a gentle stream of N_2. Samples were frozen at -20 °C until analysis by gas chromatography mass spectrometry (GCMS).

Fatty acid extraction: Tissue samples were homogenized in MilliQ water and extracted with chloroform: MeOH (2:1 v:v) spiked with 10 µg/mL pentadecanoic acid as an internal standard. Next, samples were esterified 0.5 N methanolic HCl (Sigma Aldrich, St. Louis, MO) at 65 °C for 1.5. Twenty µL of the homogenate was removed for protein quantification using a bicinchoninic acid assay (BCA) kit (ThermoFisher). Each replicate consisted of pooled extract from 3 flies.

Gas chromatography mass spectrometry (GCMS)

GCMS analysis was performed on a 7820A GC system equipped with a 5975 Mass Selective Detector (Agilent Technologies, Inc., Santa Clara, CA, USA) and a HP-5ms column ((5%-Phenyl)-methylpolysiloxane, 30 m length, 250 µm ID, 0.25 µm film thickness; Agilent Technologies, Inc.). Electron ionization (EI) energy was set at 70 eV. One microliter of the sample was injected in splitless mode and analyzed with helium flow at 1 mL/ min. The following parameters were used for fatty acids (FA): the oven was initially set at 50 °C for 2 min, increased to 90 °C at a rate of 20 °C/min and held at 90 °C for 1 min, increased to 280 °C at a rate of 5 °C/min and held at 280 °C for 2 min. For cuticular hydrocarbon (CHC) analysis, the oven was initially set at 40 °C for 3 min, increased to 200 °C at a rate of 35 °C/ min, increased

to 280 °C at a rate of 20 °C/ min, and held at 280 °C for 15 min. The MS was set to detect from *m/z* 33 to 500. Data were analyzed using MSD ChemStation (Agilent Technologies, Inc.).

Individual FA species were identified on the basis of retention time and characteristic fragmentation patterns compared to that of standards in the National Institute of Standards and Technology database (NIST 98). The area under the peak of each FA was integrated, normalized to the area of the spiked standard, and summed to determine total FA levels. To analyze changes in length between experimental and control conditions, the intensity of individual FA signals was normalized to the total peak area of all FA peaks, generating proportional values for each FA. To eliminate multi-collinearity, logcontrasts were calculated for each FA peak using the following formula:

$$logcontrast\ FA_n = \log \frac{prop(FA_n)}{prop(FA_{C17:0})}$$

where *n* represents one of the fatty acid species. A minor saturated C17 fatty acid component was used as the divisor. Relative FA abundances were pooled according to carbon chain length (C12, C14, C16, C18, C20) or to double bond number (0, 1, or 2) and analyzed by simple linear regression.

For CHC analysis, the abundance of each CHC was quantified by normalizing the area under each CHC peak to the area of the hexacosane signal using home built peak selection software (personal correspondence, Dr. Scott Pletcher, Univ. of Michigan). To calculate total CHC levels, the normalized peak area for each CHC species was summed.

References

Callahan BJ, McMurdie PJ, Rosen MJ, Han AW, Johnson AJ, Holmes SP (2016). DADA2: High-resolution sample inference from Illumina amplicon data. *Nat Methods* **13:** 581-583.

Carson HL (1987). High fitness of heterokaryotypic individuals segregating naturally within a long-standing laboratory population of *Drosophila silvestris*. *Genetics* **116:** 415-422.

Love MI, Huber W, Anders S (2014). Moderated estimation of fold change and dispersion for RNA-seq data with DESeq2. *Genome Biol* **15:** 550.

McMurdie PJ, Holmes S (2013). phyloseq: An R Package for Reproducible Interactive Analysis and Graphics of Microbiome Census Data. *PLOS ONE* **8:** e61217.

Nilsson RH, Larsson KH, Taylor AFS, Bengtsson-Palme J, Jeppesen TS, Schigel D *et al* (2019). The UNITE database for molecular identification of fungi: handling dark taxa and parallel taxonomic classifications. *Nucleic Acids Res* **47:** D259-d264.

Parada AE, Needham DM, Fuhrman JA (2016). Every base matters: assessing small subunit rRNA primers for marine microbiomes with mock communities, time series and global field samples. *Environ Microbiol* **18:** 1403-1414.

Quast C, Pruesse E, Yilmaz P, Gerken J, Schweer T, Yarza P *et al* (2013). The SILVA ribosomal RNA gene database project: improved data processing and web-based tools. *Nucleic Acids Res* **41:** D590-D596.

Robert V, Vu D, Amor AB, van de Wiele N, Brouwer C, Jabas B *et al* (2013). MycoBank gearing up for new horizons. *IMA Fungus* **4:** 371-379.

Schloss PD, Westcott SL, Ryabin T, Hall JR, Hartmann M, Hollister EB *et al* (2009). Introducing mothur: open-source, platform-independent, community-supported software for describing and comparing microbial communities. *Appl Environ Microbiol* **75:** 7537-7541.

White TJ, Bruns T, Lee S, Taylor JW (1990). Amplification and direct sequencing of fungal ribosomal RNA genes for phylogenetics. In: Innis MA, Gelfand DH, Sninsky JJ, White TJ (eds). *PCR protocols: a guide to methods and applications*. Academic Press, Inc.: New York, NY. pp 315-322.

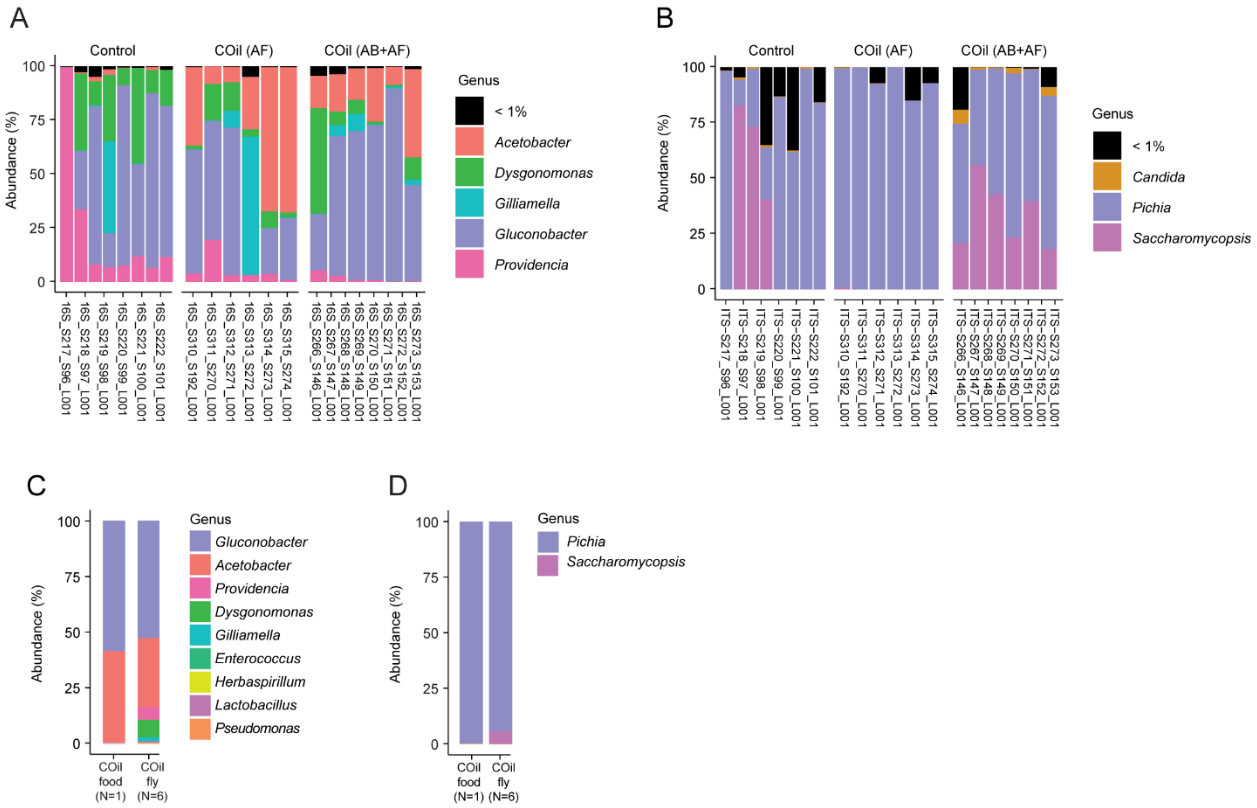

Supplementary Figure 1. Relative abundance plots for the 10 most abundant microbial genera found in individual control flies and fly food.

A. Bacterial profiles of individual control flies based on 16S rRNA amplicon sequencing (N=6 per control group).

B. Fungal profiles of individual control flies based on ITS amplicon sequencing (N=6 per control group).

C. Bacterial profiles of fresh control oil (COil) food that was not exposed to flies (N=1) and control flies raised on COil food (average of 6 flies).

D. Fungal profiles of fresh control oil (COil) food that was not exposed to flies (N=1) and control flies raised on COil food (average of 6 flies).

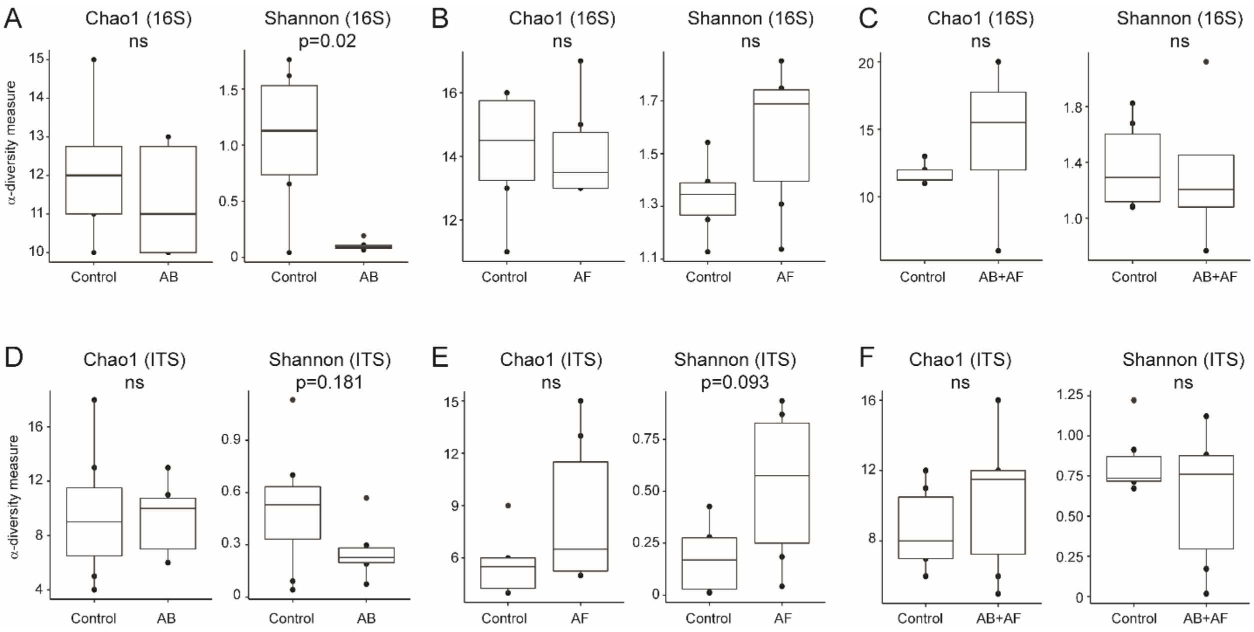

Supplementary Figure 2.

A – F. Chao1 and Shannon α-diversity indices of bacterial (top row) and fungal communities (bottom row) in control and antimicrobial-treated flies. α-diversity was compared using a Wilcoxon rank-sum test, N=6-8 for all treatments; ns: not significant (p>0.05).

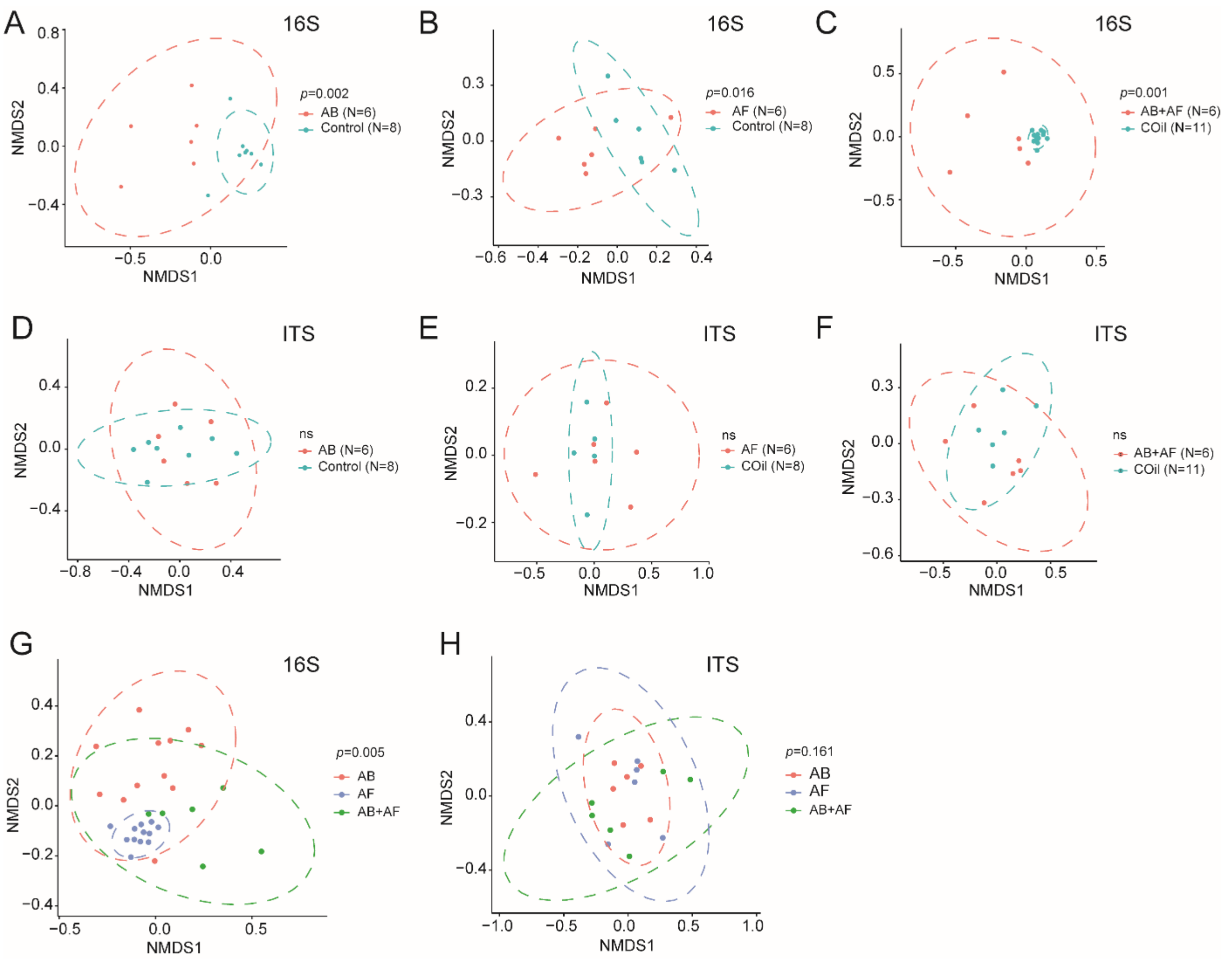

Supplementary Figure 3. Non-multidimensional scaling plots (NMDS) based on Jaccard distances) of OTUs (grouped at 97% similarity level) from control and experimental treatments. Ellipses represent significance at 0.05 confidence.

A – C. Bacterial community profiles change in response to AB, AF, or AB+AF treatments compared to respective controls (ANOSIM).

D – F. Fungal composition profiles remain stable compared to controls following AB, AF, or AB+AF treatment (all treatments $p>0.05$, ANOSIM).

G. Flies from antibacterial (AB; N=11), antifungal (AF; N=12), and AB+AF (N=6) experimental treatments contain distinct bacterial communities (ANOSIM).

H. There was no significant separation in fungal composition between the AB, AF, and AB+AF treatment groups (ANOSIM, N=6 per sex).

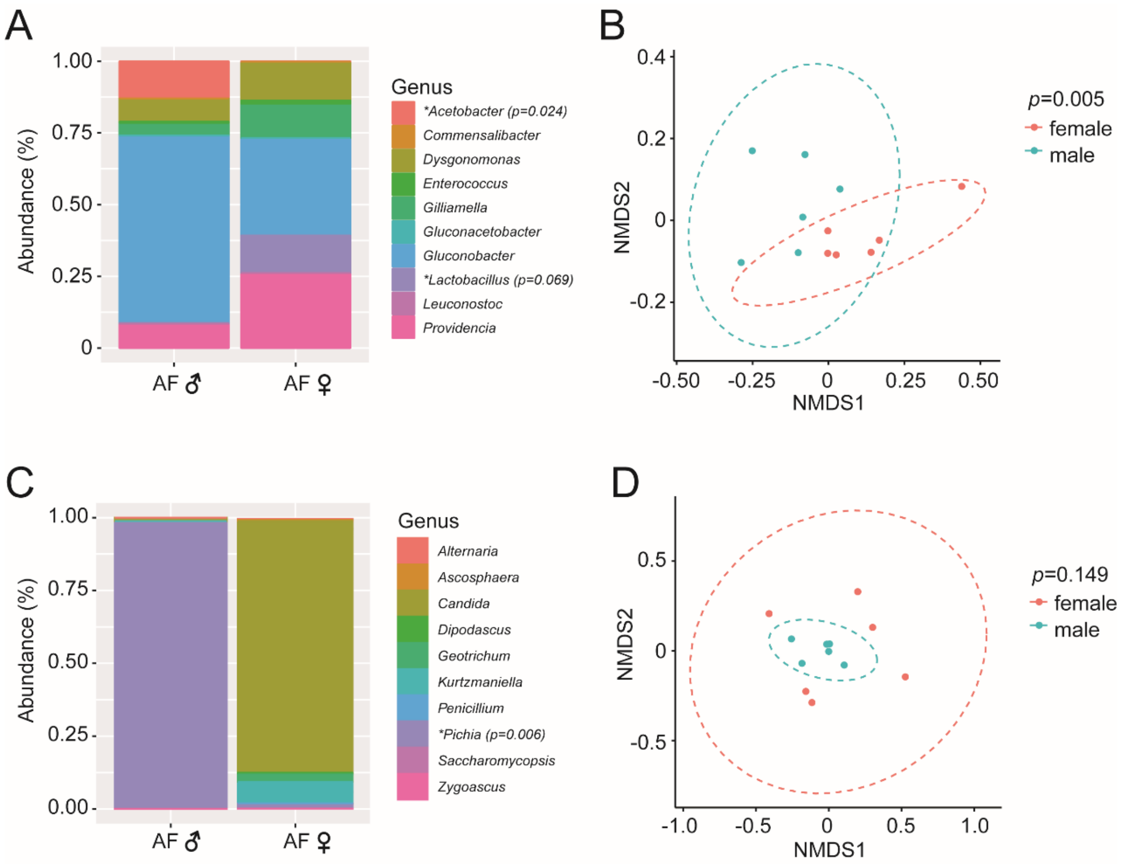

Supplementary Figure 4.

Comparison of male and female microbiome 16S rRNA and ITS high throughput sequencing amplicon profiles following antifungal treatment. Samples are comprised of flies that were treated for 21 or 35 days.

A. Scaled relative abundance plots for the 10 most abundant bacterial genera of males and females following AF treatment (N=6 per sex). The *p* values were determined using univariate multiple testing with an F test; *: abundance is significantly different between control and treatment conditions.

B. Non-multidimensional scaling plots (NMDS; based on Jaccard distances) of OTUs reveal distinct bacterial communities in males and females following AF treatment (ANOSIM, N=6 per sex). Ellipses represent significance at 0.05 confidence.

C. Scaled relative abundance plots for the 10 most abundant fungal genera of males and females following AF treatment (N=6 per sex). The *p* values were determined using univariate multiple testing with an F test; *: significantly different between control and treatment conditions.

D. Non-multidimensional scaling plots (NMDS; based on Jaccard distances) of OTUs show no significant separation in fungal composition between males and females (ANOSIM, N=6 per sex). Ellipses represent significance at 0.05 confidence.

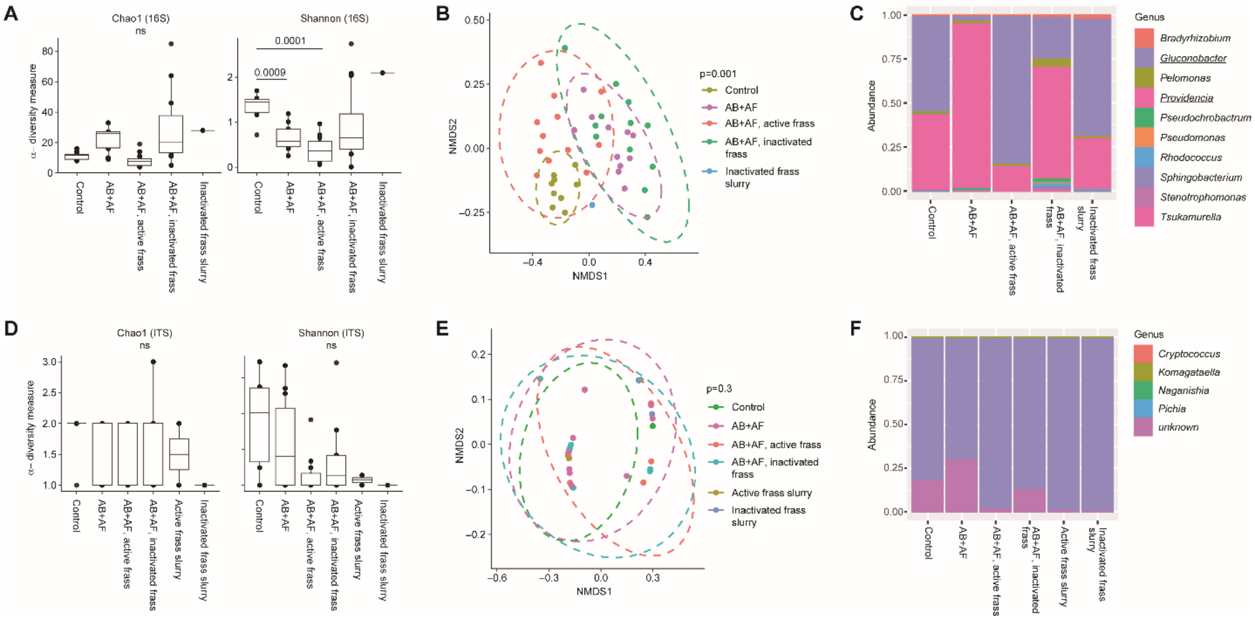

Supplementary Figure 5.

Analysis of 16S rRNA and ITS high throughput sequencing amplicon profiles of control flies, AB+AF-treated females, and antibacterial and antifungal (AB+AF)-treated females following inoculation with active or inactivated frass from control flies (N=10 per fly group; N=1 for slurry).

A. Chao1 and Shannon α-diversity indices of bacterial communities in flies and frass slurry. The α-diversity measures were compared using a Wilcoxon rank-sum test; ns: not significant ($p>0.05$).

B. Non-multidimensional scaling plots (NMDS; based on Jaccard distances) of OTUs reveal that the bacterial profiles of control flies and AB+AF flies treated with active frass are more similar to each other compared to other treatments (ANOSIM). Ellipses represent significance at 0.05 confidence.

C. Scaled relative abundance plots for the 10 most abundant bacterial genera. Underlined taxa are *not* significantly different between Control and AB+AF flies treated with active frass.

D. Chao1 and Shannon α-diversity indices of fungal communities in flies and frass slurry. The α-diversity measures were compared using a Wilcoxon rank-sum test; ns: not significant ($p>0.05$).

E. Non-multidimensional scaling plots (NMDS; based on Jaccard distances) of OTUs reveal that AB+AF treatment and various frass inoculations did not significantly alter the fungal composition of flies compared to controls (ANOSIM). Ellipses represent significance at 0.05 confidence.

F. Scaled relative abundance plots for the 10 most abundant fungal genera.

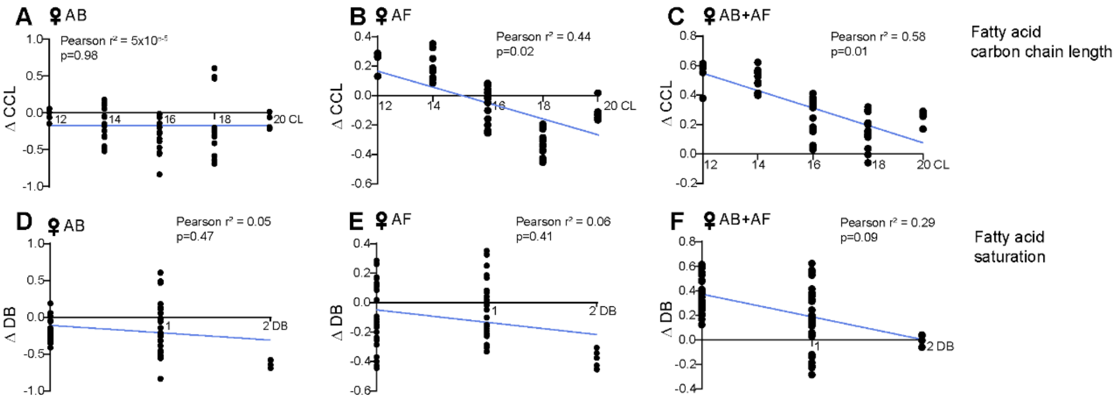

Supplementary Figure 6.

Influence of microbiome composition on female whole body fatty acid carbon chain length and degree of saturation. The Pearson r^2 value and simple linear regression line are provided; N=3-5.

A – C. Change in whole body fatty acid carbon chain lengths (CCL) in antibacterial- (AB), antifungal- (AF), or AB+AF treated-females compared to controls.

D – F. Change in whole body fatty acid saturation levels in treated females compared to controls; DB: double bond number.

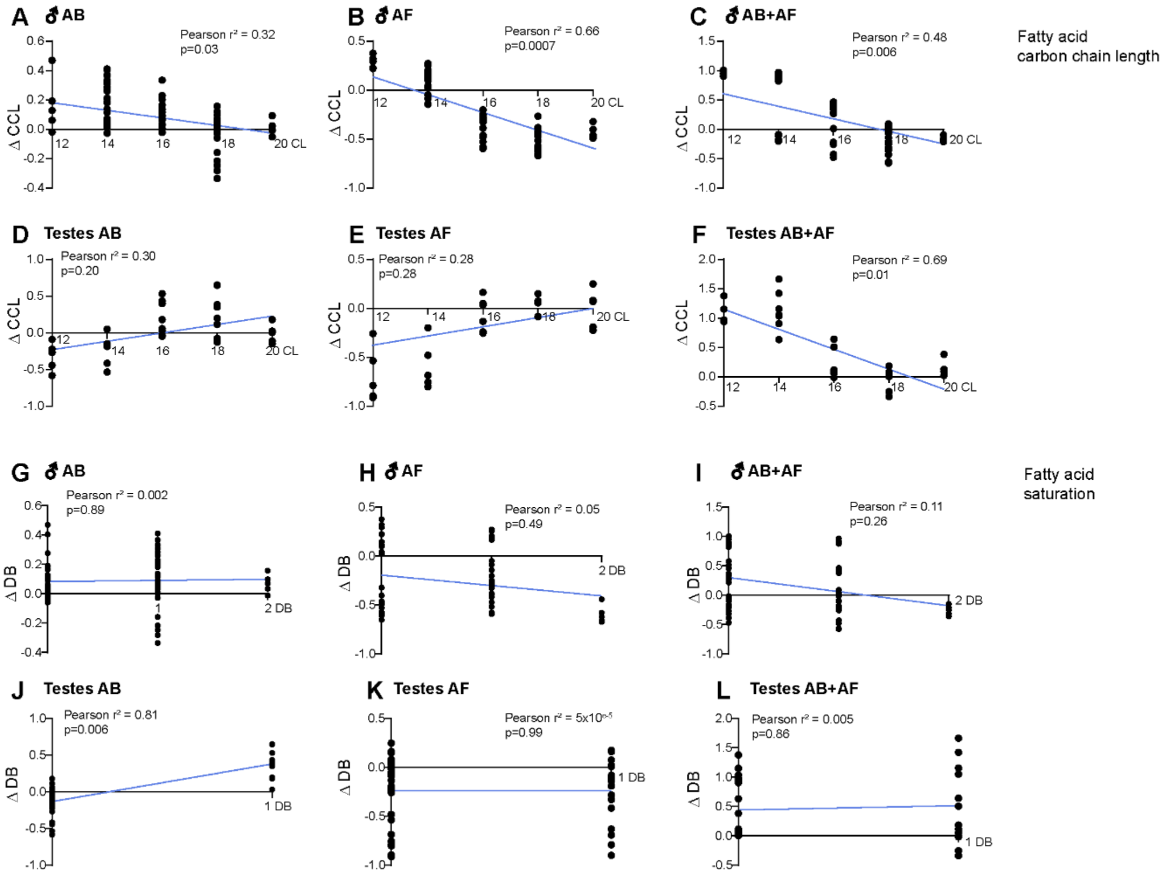

Supplementary Figure 7.

Influence of microbiome composition on male whole body and testes fatty acid carbon chain length and degree of saturation. The Pearson r^2 value and simple linear regression line are provided; N=3-5.

A – C. Change in whole body fatty acid carbon chain lengths (CCL) in antibacterial (AB), antifungal (AF), or AB+AF treated-males compared to controls.

D – F. Change in testes fatty acid carbon chain lengths (CCL) in antibacterial- (AB), antifungal- (AF), or AB+AF-treated males compared to controls.

G – I. Change in whole body fatty acid saturation levels in treated males compared to controls; DB: double bond number.

J – L. Change in testes fatty acid saturation levels in treated males compared to controls; DB: double bond number.

Supplementary Table 1. Parameter estimates for negative binomial regression analysis of bacterial colony forming units following antimicrobial treatments.

Treatment	Coefficient[1]	SE[2]	Z value	95% CI[3]	*p*
Control vs. AB	-2.9899	0.2436	-12.27	-3.47, -2.51	<0.0001
COil vs. AF	0.5409	0.6464	0.837	-0.73, 1.81	ns
COil vs. AB+AF	-2.5123	0.6586	-3.815	-3.80, -1.22	<0.0001
AF vs. AB+AF	-3.0532	0.6582	-4.639	-4.34, -1.76	<0.0001

[1]Estimated negative binomial regression coefficient.
[2]Standard error.
[3]95% confidence interval; ns; not significant.

Supplementary Table 2. Parameter estimates for negative binomial regression analysis of fungal colony forming units following antimicrobial treatments.

Treatment	Coefficient[1]	SE[2]	Z value	95% CI[3]	*p*
Control vs. AB	1.7803	0.4737	3.758	0.85, 2.71	<0.0001
COil vs. AF	-5.5294	0.6384	-8.661	-6.78, -4.28	<0.0001
COil vs. AB+AF	-2.7994	0.5018	-5.579	-3.78, -1.82	<0.0001
AF vs. AB+AF	2.73E+00	6.46E-01	4.224	1.46, 3.99	<0.0001

[1]Estimated negative binomial regression coefficient.
[2]Standard error.
[3]95% confidence interval.

Supplementary Table 3. Parameter estimates for negative binomial regression analysis of egg counts following antimicrobial treatment and frass transplants from control flies.

**Treatment**[1]	**Coefficient**[2]	**SE**[3]	**Z value**	**95% CI**[4]	***p***
Control vs. AB+AF	-4.3287	1.0116	-4.279	-6.31, -2.35	<0.0001
Control vs. Active FT	-1.2196	0.7499	-1.626	-2.69, 0.25	ns
COil vs. Inactivated FT	-2.9695	0.7375	-4.027	-4.41, -1.52	<0.0001
AB+AF vs. Active FT	-3.1091	0.9737	-3.193	-5.02, -1.20	<0.001
Inactivated FT vs. Active FT	-1.7499	0.6846	-2.556	-3.09, -0.41	<0.01

[1]AB: antibiotic treated; AF: antifungal treated; COil: control oil; FT: fecal transfer.
[2]Estimated negative binomial regression coefficient.
[3]Standard error; ns: not significant.
[4]95% confidence interval.

Supplementary Table 4. Parameter estimates for negative binomial regression analysis of egg counts following co-housing treatment.

**Treatment**[1]	**Coefficient**[2]	**SE**[3]	**Z value**	**95% CI**[4]	***p***
Control vs. AB+AF	-4.585	1.797	-2.551	-8.11, -1.06	<0.01

[1]AB+AF: antibiotic and antifungal treated.
[2]Estimated negative binomial regression coefficient.
[3]Standard error.
[4]95% confidence interval.

Supplementary Table 5. Parameter estimates for negative binomial regression analysis of egg counts following frass transplant from control or treated flies.

**Treatment**[1]	**Coefficient**[2]	**SE**[3]	**Z value**	**95% CI**[4]	***p***
Control vs. AB	-1.6644	0.6976	-2.386	-3.03, -0.30	<0.01
Control vs. AF	-1.4697	0.8034	-1.829	-0.30, 0.11	<0.05

[1]AB: antibiotic-treated; AF: antifungal-treated.
[2]Estimated negative binomial regression coefficient.
[3]Standard error.
[4]95% confidence interval.

Supplementary Table 6. Parameter estimates for negative binomial regression analysis of egg counts following antifungal, antibacterial, or antifungal and antimicrobial treatment.

**Treatment[1]**	**Coefficient[2]**	**SE[3]**	**Z value**	**95% CI[4]**	***p***
Control vs. AB	-0.5899	0.5231	-1.128	-1.62, 0.44	ns
COil vs. AF	-1.0636	0.3832	-2.775	-1.81, -0.31	<0.001
COil vs. AB+AF	-3.6868	0.4528	-8.142	-4.57, -2.80	<0.0001
AF vs. AB+AF	-2.6232	0.4959	-5.290	-3.60, -1.65	<0.0001
AB vs. AB+AF	-2.3311	0.6855	-3.401	-3.67, -0.99	<0.0001

[1]AB: antibiotic-treated; AF: antifungal-treated.
[2]Estimated negative binomial regression coefficient.
[3]Standard error.
[4]95% confidence interval; ns: not significant.

Supplementary Table 7. Female cuticular hydrocarbon levels**.**

**Treatment**	**Mean**	**SD[1]**	**95% CI[2]**	***p*[3]**	***g*[4]**
Control	0.863	0.122	0.56, 1.17	0.20	2.14
AB	1.125	0.123	0.82, 1.43		
COil	2.245	0.354	1.37, 3.12	0.10	5.85
AF	4.630	0.455	3.50, 5.76		
COil	0.758	0.446	-0.35, 1.87	>0.99	0.003
AB+AF	0.759	0.422	-0.29, 1.81		

[1]Standard deviation; [2]Lower and upper confidence interval of mean; [3]Two-tailed Mann-Whitney U test; [4]Hedges' *g* effect size

Supplementary Table 8. Male cuticular hydrocarbon levels.

**Treatment**	**Mean**	**SD[1]**	**95% CI[2]**	***p*[3]**	***g*[4]**
Control	1.097	0.165	0.69, 1.51	>0.99	0.07
AB	1.078	0.348	0.21, 1.94		
COil	1.854	0.718	1.10, 2.61	0.0238	2.96
AF	4.000	0.745	2.15, 5.85		
COil	0.872	0.756	-1.01, 2.75	0.20	1.18
AB+AF	0.235	0.090	0.01, 0.46		

[1]Standard deviation; [2]Lower and upper confidence interval of mean; [3]Two-tailed Mann-Whitney U test; [4]Hedges' *g* effect size

Supplementary Table 9. Female fatty acid levels: whole body.

Treatment	Mean	SD[1]	95% CI[2]	*p*[3]	*g*[4]
Control	70.02	20.94	48.04, 92.00	0.0381	1.48
AB	44.10	9.23	29.42, 58.79		
COil	47.32	13.56	30.48, 64.17	0.0159	3.47
AF	100.7	17.48	72.92, 128.60		
COil	63.43	4.94	55.57, 71.29	0.0317	2.71
AB+AF	46.07	7.80	36.39, 55.76		

[1]Standard deviation; [2]Lower and upper confidence interval of mean; [3]Two-tailed Mann-Whitney U test; [4]Hedges' *g* effect size

Supplementary Table 10. Male fatty acid levels: whole body.

Treatment	Mean	SD[1]	95% CI[2]	*p*[3]	*g*[4]
Control	30.75	8.67	19.99, 41.51	0.69	0.81
AB	39.52	12.69	23.76, 55.28		
COil	22.98	4.76	17.08, 28.89	0.0079	5.65
AF	56.87	7.04	48.14, 65.61		
COil	25.81	1.20	24.32, 27.30	0.0079	5.04
AB+AF	78.57	14.77	60.24, 96.91		

[1]Standard deviation; [2]Lower and upper confidence interval of mean; [3]Two-tailed Mann-Whitney U test; [4]Hedges' *g* effect size

Supplementary Table 11. Male fatty acid levels: testes.

Treatment	Mean	SD[1]	95% CI[2]	*p*[3]	*g*[4]
Control	4.99	0.77	4.03, 5.94	0.0317	2.08
AB	3.47	0.69	2.61, 4.32		
COil	6.35	0.27	5.67, 7.03	0.857	0.52
AF	6.67	0.75	5.47, 7.87		
COil	3.36	0.27	3.02, 3.70	0.0079	5.95
AB+AF	6.02	0.61	5.06, 6.99		

[1]Standard deviation; [2]Lower and upper confidence interval of mean; [3]Two-tailed Mann-Whitney U test; [4]Hedges' *g* effect size
